# Sources of contamination, prevalence, and antimicrobial resistance of thermophilic *Campylobacter* isolated from turkeys

**DOI:** 10.14202/vetworld.2018.1074-1081

**Published:** 2018-08-07

**Authors:** Radia Bouhamed, Leila Bouayad, Sara Messad, Safia Zenia, Malek Naïm, Taha-Mossadak Hamdi

**Affiliations:** 1 Laboratory of Food Hygiene and Quality Insurance System, High National Veterinary School, Rue Issad Abbes, 16111 El Alia, Oued Smar, Algiers, Algeria; 2 Department of Microbiology, Central Military Hospital, 16050 Kouba, Algiers, Algeria

**Keywords:** antimicrobial resistance, farm, slaughterhouse, Thermophilic *Campylobacter*, turkey

## Abstract

**Aim:**

Sources of contamination, prevalence, and antimicrobial susceptibility of thermophilic *Campylobacter* isolated from turkey samples were determined.

**Materials and Methods:**

A total of 300 samples were collected from 3 farms (fecal droppings) and 4 poultry slaughterhouses (neck skins and ceca) located in the middle area of Algeria (Algiers, Boumerdès, and Bouira). After detection, an antibiogram was realized only for slaughterhouses samples.

**Results:**

Samples from cecum (90.0%, 90/100; 95% confidence interval (CI)=84.1-95.9%), fecal dropping (68.0%, 68/100; 95% CI=58.9–77.1%), and neck skin (55.0%, 55/100; 95% CI=45.2–64.8%) were positive for thermophilic *Campylobacter* (p<0.05). Contamination rate of turkey carcasses was higher in modern slaughterhouse (96.7%) than in traditional slaughterhouses (37.1%) (p<0.05). Isolated strains were resistant to nalidixic acid (NA) (87.5%), tetracycline (TE) (81.3%), ciprofloxacin (CIP) (75.0%), ampicillin (AM) (65.6%), and erythromycin (25.0%) (p<0.05). 96.9% (124/128) of the isolates were multiresistant and 18 drug resistance patterns were registered. The predominant one (43.0%) was AM, NA, CIP, and TE.

**Conclusions:**

Potential sources of contamination of this fastidious bacterium were noticed in farms and slaughterhouses. Modern slaughterhouse allowed contamination of turkey carcasses more than a traditional slaughterhouse. However, the scalding step could not represent a source of contamination. The most tested strains exhibited resistance to erythromycin and/or CIP. It is worrisome because these molecules are considered as first-choice antibiotics for human campylobacteriosis.

## Introduction

*Campylobacter* is considered worldwide as the major cause of gastroenteritis in humans [1]. In 2010, 109,700 fatal cases worldwide were reported. The disease rate in developing countries is 400 to 600 per 100.000 among children <5 years of age. For developed countries, the disease rate is 300 per 100.000. In both developing and developed countries, rates in the general population are estimated at 90 per 100.000 [2]. Human campylobacteriosis occurs mainly following consumption of contaminated raw or undercooked food or contaminated water [1]. Furthermore, due to the gut colonization by thermophilic *Campylobacter* of animals intended for human consumption, meat contamination has mainly a digestive origin, and it occurs during slaughtering [1,3].

However, among the entire foodstuff, poultry meat notably broiler and turkey are considered to be the main vehicle of thermophilic *Campylobacter* to human [4]. A low infectious dose is enough to cause ordinary *Campylobacter* enteritis which frequently progresses to hemorrhagic enteritis and sometimes even to a Guillain-Barre syndrome [1,3]. In general, the patient eventually heals without resorting to antibiotic treatment, but in severe cases, antibiotic treatment is needed [5]. Thermophilic *Campylobacter* both in animals and humans have acquired over time resistance to various antibiotics, including erythromycin, and ciprofloxacin (CIP), the major molecules for the treatment of *Campylobacter* infection [6]. Thus, the presence of *Campylobacter* strains resistant to ­antibiotics in foodstuffs of animal origin represents a sig­­nificant threat to public health [5]. In Algeria, a previous study reported that thermophilic *Campylobacter* was considered as a cause of human gastroenteritis. Indeed, they were isolated from human fecal samples with a rate of 17.7% [7]. In developed countries, thermophilic *Campylobacter* isolation rates ranged from 55 to 77% in turkey samples [8,9].

To the best of our knowledge, there are no published data on the presence of thermophilic *Campylobacter* in Algerian turkey samples, even if turkey meat is frequently consumed by our population. Therefore, the current study was carried out to determine sources of contamination, the incidence of thermophilic *Campylobacter* on turkey in both poultry farms and poultry slaughterhouses, and to study the antibiotic susceptibility of *Campylobacter* strains isolated from poultry slaughterhouses samples.

## Materials and Methods

### Ethical approval

In this study, we used turkey droppings, samples from cecum and neck skin of turkey carcasses. Therefore, no ethical approval was needed.

### Samples collection

Before each sampling, a questionnaire was filled out through the information provided by the owner and in some cases by the veterinarian of the establishment. 300 samples were collected aseptically and randomly from 7 establishments located in the region of Algiers: 3 farms and 4 poultry slaughterhouses. This allowed us to have samples from various locations and regions. During the end of the rearing period, a total of 100 samples of fecal droppings were collected 1-2 weeks before removal in three turkey farms (A, B, and C). In addition, 100 samples each of neck skins and ceca were collected just after turkey’s evisceration within 4 poultry slaughterhouses (three traditional [A, B, and C] and one modern [D]). With a livestock capacity ranging from 800 to 900 subjects, the visited farms were located in rural areas including subjects delivered from the same hatchery and reared in a single band until slaughter. The visited slaughterhouses were located in urban or industrial areas, and their capacity ranged from 300 to 700 birds per week.

All the samples were taken early in the morning within 2 h. 1-2 weeks before slaughter, fresh fecal samples were collected from litters using sterile spatulas. We paid attention to collect them quickly and aseptically just after their emission on the ground without any trace of litter or urine. At present, there is not an accepted standard method for the detection and isolation of *Campylobacter* spp. at farm level [10]. We decided not to choose cloacal swabs because we followed the OIE manual which recommends to collect freshly voided feces (>10^6^ UFC/g) for reliable detection of *Campylobacter* by culture [11,12]. Furthermore, according to some authors, isolation from fecal samples give better results than isolation from cloacal swabs [13]. During the slaughtering process, just after the evisceration step, neck skins and ceca were aseptically collected. Fecal dropping and cecum samples were put in separate sterile universal plastic sample containers, while neck skins were placed in separate sterile plastic bags. After that, all the samples were placed inside a cool box and transported immediately to the Department of Microbiology of the Central Military Hospital of Algiers where they were processed within 2-4 h.

### Detection of thermophilic Campylobacter

All the *Campylobacter* cultures (isolation, identification, and antimicrobial susceptibility testing) were obtained with the microaerophilic generators GENbagmicroaer (bioMérieux) (5% O_2_, 10% CO_2_, and 85% N_2_).

### Identification of thermophilic Campylobacter

From each sample of feces or cecal content, 1g was inoculated into 9 ml of sterile saline (0.9% NaCl, w/v) and homogenized. For neck skins, 10 g of each neck skin was added in 90 ml of Preston broth (Oxoid) with 5% horse blood (IPA: Institut Pasteur d’Algérie) and incubated at 42°C for 24 h. An agar Campylosel ready to use (bioMérieux) for droppings samples, and a Butzler agar (Oxoid) with 5% horse blood (IPA) for neck skin samples and cecal contents, were seeded and incubated at 42°C for 48 h in the microaerophilic atmosphere [14].

### Confirmation of the suspected colonies

Once purified onto Columbia agar (Bio Rad) with 5% horse blood (IPA), identification of *Campylobacter* strains was performed using the standard tests: Gram stain, motility, catalase, and oxidase reactions, growth at 25°C and aerobic growth. Suspected colonies of *Campylobacter* were subjected to confirmation by studying biochemical tests on triple sugar iron agar (IPA) and by testing their sensitivity to nalidixic acid (NA) (30 µg) and cephalothin (KF) (30 µg) [11,15]. After that, only one strain from each *Campylobacter*-positive sample was selected for susceptibility testing.

### Antimicrobial susceptibility testing

Determination of antibiotic susceptibility of *Campylobacter* was realized only for strains isolated from samples that were collected from poultry slaughterhouses, following the disk diffusion method as recommended by the antibiogram committee of the “French Society of Microbiology” [16]. In addition to NA and KF, the tested antibiotics were: Ampicillin (AM) (10 µg), gentamicin (15 µg) (GM), erythromycin (15 UI) (E), CIP (5 µg), tetracycline (30 UI) (TE), and chloramphenicol (30 µg) (C). From a pure culture of 18-24 h of incubation, a bacterial suspension of 0.5 McFarland opacity was prepared and diluted 1:10. After seeding by swabbing on Mueller-Hinton agar (Bio-Rad) containing 5% horse blood (IPA), and applying discs of antibiotics (bioMérieux), the plates were in the microaerophilic atmosphere during 24 h at 37°C. The diameters of inhibition zones were measured using a metal caliper. For quality control, the reference strains *Escherichia coli* ATCC 25922 and *Staphylococcus aureus* ATCC 25923 were used.

### Statistical analysis

Chi-square and Fisher’s exact tests were performed to compare the results of the tested samples and the antimicrobial susceptibility testing. The difference was significant when the p<0.05. Furthermore, 95% confidence interval (95% CI) was also determined for contamination and resistance rates.

## Results

### Sources of contamination

In all the establishments a host of risk factors were observed. The results of farm and slaughterhouse surveys are reported in Table-1. Most farms were mixed houses (66.7%) where fresh litter was used (66.7%), access of wild or domestic animals in the houses was not controlled (100.0%), turkey feces were used as manure to fertilize crops (100.0%), and drinking water was dirty and contained feathers (100.0%). In slaughterhouses, several sources of contamination such as the absence of maintaining forward movement (0.0%), fixed station (0.0%), sterilization of the slaughtering equipment (0.0%), cleaning and/or disinfection protocol (0.0%), and presence of dirty uniforms (75.0%) were observed.

**Table-1:** Potential sources of *Campylobacter* transmission in farms and slaughterhouses.

General characteristics	Farms			Total n (%)

	A	B	C	
Mixed flock (broiler+turkey)	+	-	+	2/3 (66.7)
Other livestock	Rabbits and bees	cattle	-	2/3 (66.7)
Other animals (dogs, cats, wild birds, and rodents)	+^a^	+	+	3/3 (100.0)
Insects	Flies	Flies	-	2/3 (66.7)
Litter	Spent (moist)	Fresh (dry)	Fresh (dry)	2/3 (66.7)^b^
Water quality control	-	-	-	0/3 (0.0)
Drinking water	Dirty	Dirty	Dirty	3/3 (100.0)
Pest control	Rats and insects	-	Rats	2/3 (66.7)
Cleaning and/or disinfection protocol	-	-	-	0/3 (0.0)
Using turkey feces as manure	+	+	+	3/3 (100.0)

	**Slaughterhouses**			

	**A**	**B+C**	**D**	

Respect of transport conditions (from flock to slaughterhouse)	-	-	-	0/4 (0.0)
Respect of feed withdrawal+rest period	-	+	-	2/4 (50.0)
Mixed slaughterhouse (broiler+turkey)	+	-	+	2/4 (50.0)
Slaughtering process	Manual	Manual	Industrial	3/4 (75.0)^c^
Maintaining forward movement	-	-	-	0/4 (0.0)
Fixed station	-	-	-	0/4 (0.0)
Respect of the scalding water temperature	-	-	+	1/4 (25.0)
Sterilization of the slaughtering equipment	-	-	-	0/4 (0.0)
Cleaning and/or disinfection protocol	-	-	+	0/4 (0.0)
Worker’s uniform	Dirty	Dirty	Clean	1/4 (25.0) 3/4 (75.0)

a Plus wild boar,

b Fresh litter (dry),

c Manual

### Detection of thermophilic Campylobacter

Thermophilic *Campylobacter* strains were isolated with high prevalence in all the sampled turkey flocks (71.0% and 213/300) in both farms (68.0% and 68/100) and slaughter facilities (72.5% and 145/200) (Table-2). In total, they were detected in 90.0% (95% CI = 84.1-95.9%) of cecal contents, 68.0% (95% CI = 58.9-77.1%) of fecal droppings, and 55.0% (95% CI = 45.2-64.8%) of neck skins (Table-2). The difference between these results was statistically significant (p<0.05). Furthermore, for neck skin samples, the overall prevalence of thermophilic *Campylobacter* isolated from traditional slaughterhouses (37.1%, 26/70; 95% CI=25.8-48.5%) was lower than the one registered in modern slaughterhouse (96.7%, 29/30; 95% CI=82.8-99.9%) (p<0.05). However, thermophilic *Campylobacter* was isolated with high rates from cecal contents in both traditional and modern slaughterhouses as a whole (88.6%, 62/70 vs. 93.3%, 28/30; p>0.05).

**Table-2:** Prevalence of thermophilic *Campylobacter* in the visited farms and poultry slaughterhouses.

Farms		Slaughterhouses		
	
Flock	Fecal droppings Number /examined samples (%)	Flock	Cecal content Number /examined samples (%)	Neck skin Number/examined samples (%)
A	29/35 (82.9)	A^a^	16/20 (80.0)	6/20 (30.0)
B	17/32 (53.1)	B^a^	19/20 (95.0)	13/20 (65.0)
C	22/33 (66.7)	C^a^	27/30 (90.0)	7/30 (23.3)
Total	68/100 (68.0)	D	28/30 (93.3)	29/30 (96.7)
		Total	90/100 (90.0)	55/100 (55.0)

a Significant difference (p<0.05) between the results of cecal contents and neck skins

### Antimicrobial susceptibility testing

Antimicrobial susceptibility testing was studied for 128 of 145 isolated strains from poultry slaughterhouses because 17 thermophilic *Campylobacter* strains were impossible to re-streak (viable but non-cultivable form). 87.5% (n=112; 95% CI=81.8-93.2) strains were resistant to NA, 81.3% (n=104; 95% CI=74.5-88.0) to TE, 75.0% (n=96; 95% CI=67.5-82.5) to CIP, 65.6% to AM (n=84; 95% CI=57.4-73.9), and 25.0% (n=32; 95% CI=17.5 32.5) to E, and no resistance was recorded for GM and chloramphenicol (C) (0.0%) (p<0.05) (Table 3). The study of the antimicrobial susceptibility of TTC according to the type of samples revealed that the difference between the rates of each tested antibiotic for strains isolated from cecal contents and neck skins was not statistically significant (p>0.05) (Table-3). Furthermore, rates of resistance to NA, E, CIP, TE, and AM between all the visited slaughterhouses (A, B, C, and D) were significantly different (Table-4). Multidrug-resistant *Campylobacter* isolates were common. All the tested strains were resistant to at least one antibiotic (100.0%), 124 of 128 tested strains (96.9%) were multidrug-resistant. 17.2% (n=22) isolates were resistant to two antibiotics, 25.0% (n=32) to three antibiotics, 51.6% (n=66) to four antibiotics, and 3.1% (n=4) to five antibiotics. Furthermore, 18 drug resistance patterns were identified, and the most prevalent multiple resistance profiles were observed for 55 isolates (43.0%) and included AM, NA, TE, and CIP. 17 antimicrobial resistance patterns for cecal contents isolates and 9 antimicrobial resistance patterns of thermophilic *Campylobacter* strains isolated from neck skins were noticed. Resistance to CIP and/or E was found in 85.9% of the isolates that were multiresistant (n=110). Results of resistance patterns of thermophilic *Campylobacter* isolates (n=124) that were resistant to two or more antibiotics are reported in Table-5.

**Table-3:** Antimicrobial resistance rates of thermophilic*Campylobacter* strains according to the type of samples.

Tested antibiotics^a^	All the samples (n=128)		Type of samples	
	
	n (%)	95% CI^b^	Cecal content n=81 (%)	Neck skin n=47 (%)
NA^c^	112 (87.5)	81.893.2	65 (80.3)	46 (97.9)
TE^c^	104 (81.3)	74.588.0	63 (77.8)	41 (87.2)
CIP^c^	96 (75.0)	67.582.5	59 (72.8)	37 (78.7)
AM^c^	84 (65.6)	57.473.9	52 (64.2)	32 (68.1)
E^c^	32 (25.0)	17.532.5	18 (22.2)	14 (29.8)
GM	0 (0.0)	-	0 (0.0)	0 (0.0)
C	0 (0.0)	-	0 (0.0)	0 (0.0)

a NA=Nalidixic Acid, TE=Tetracycline, CIP=Ciprofloxacin, AM=Ampicillin, E=Erythromycin, GM=Gentamicin, C=Chloramphenicol,

b 95% confidence interval,

c No significant difference (p>0.05) between the results of cecal contents and neck skins for each tested antibiotic

**Table-4:** Antimicrobial resistance rates of thermophilic*Campylobacter* strains according to the visited slaughterhouses.

Tested antibiotics^a^	A n=17 (%)	B n=29 (%)	C n=27 (%)	D n=55 (%)
NA^b^	9 (52.9)	24 (82.8)	27 (100.0)	51 (92.7)
TE^b^	15 (88.2)	14 (48.3)	21 (77.8)	54 (98.2)
CIP^b^	12 (70.6)	7 (24.1)	22 (81.5)	55 (100.0)
AM^b^	9 (52.9)	13 (44.8)	22 (81.5)	40 (72.7)
E^b^	17 (100.0)	8 (27.6)	6 (22.2)	1 (1.8)
GM	0 (0.0)	0 (0.0)	0 (0.0)	0 (0.0)
C	0 (0.0)	0 (0.0)	0 (0.0)	0 (0.0)

a NA=Nalidixic acid, TE=Tetracycline, CIP=Ciprofloxacin, AM=Ampicillin, E=Erythromycin, GM=Gentamicin, C=Chloramphenicol,

b Significant difference (p<0.05) between the results of the slaughterhouses for each tested antibiotic

**Table-5:** Resistance patterns of thermophilic*Campylobacter* strains isolated from slaughterhouses.

No. antimicrobials	Pattern^a^	Isolated strains n=124 (%)	Cecal contents n=78 (%)	Neck skins n=46 (%)
2	AM-NA	9 (7.3)	9 (11.5)	0 (0.0)
2	TE-NA	5 (4.0)	4 (5.1)	1 (2.2)
2	CIP-TE	3 (2.4)	3 (3.8)	0 (0.0)
2	CIP-NA	2 (1.6)	1 (1.3)	1 (2.2)
2	E-NA	2 (1.6)	1 (1.3)	1 (2.2)
2	AM-CIP	1 (0.8)	0 (0.0)	1 (2.2)
	Total	22 (17.7)	18 (23.1)	4 (8.7)
3	CIP-TE-NA	11 (8.9)	11 (14.1)	0 (0.0)
3	E-TE-NA	9 (7.3)	2 (2.6)	7 (15.2)
3	AM-CIP-NA	7 (5.6)	5 (6.4)	2 (4.3)
3	E-CIP-TE	2 (1.6)	2 (2.6)	0 (0.0)
3	E-CIP-NA	1 (0.8)	1 (1.3)	0 (0.0)
3	AM-E-TE	1 (0.8)	1 (1.3)	0 (0.0)
3	AM-CIP-TE	1 (0.8)	1 (1.3)	0 (0.0)
	Total	32 (25.8)	23 (29.5)	9 (19.6)
4	AM-CIP-TE-NA	55 (44.4)	28 (35.9)	27 (58.7)
4	AM-E-CIP-TE	5 (4.0)	5 (6.4)	0 (0.0)
4	E-CIP-TE-NA	5 (4.0)	1 (1.3)	4 (8.7)
4	AM-E-TE-NA	1 (0.8)	1 (1.3)	0 (0.0)
	Total	66 (53.2)	35 (44.9)	31 (67.4)
5	AM-E-CIP-TE-NA	4 (3.2)	2 (2.6)	2 (4.3)
	Total	4 (3.2)	2 (2.6)	2 (4.3)

a NA=Nalidixic acid, TE=Tetracycline, CIP=Ciprofloxacin, AM=Ampicillin, E=Erythromycin, GM=Gentamicin, C=Chloramphenicol

## Discussion

Except the influence of selective culture media and sources of contamination noted on livestock, the significant difference (p<0.05) between the rates of *Campylobacter* contamination of fecal samples and cecal contents may also depend on the sampling season. Indeed, fecal droppings were sampled in winter while cecal contents were collected in summer. The peak of contamination of poultry flocks by *Campylobacter* occurs in warmer months [17,18].

The prevalence of thermophilic *Campylobacter* in fecal droppings was in agreement with a previous study in Greece (77%) [19]. However, lower rates (56%) have been observed in the USA [9]. On turkey farms, these bacteria were present in at least half of the fecal samples; which involves a significant horizontal transmission. Indeed, in all the visited farms, we observed a host of risk factors (Table-1) described for broiler flock colonization [18] such as exposure to potential sources of the bacterium such as the presence of humans, other animals (wild and domestic animals), insects and rodents on farms, types and quality of litter, having access to outside soil, and using poultry feces as manure. Contamination from previous flocks may also represent a risk factor [18]. Knowing that to reduce the rate of *Campylobacter* in farms, a high level of biosecurity control and hygiene must be done [18] and as there was no cleaning and/or disinfection protocol in all the visited farms (Table-1), we suggested that it was possible that previous flocks contaminated the tested flocks as reported by some authors [18,20]. Giving dirty drinking water could be another risk factor. As it contained feathers and was not chlorinated, we suggested that *Campylobacter* can be present as biofilms in water as reported by some authors [18]. Furthermore, chlorinated water can reduce the risk of broiler colonization [21,22], but it was not the case for our study. In farm A, the rate of thermophilic *Campylobacter* was higher than the rates registered in farms B and C. The presence of several animal species was more detected in farm A than farms B and C. They not only included dogs, cats, wild birds, and rodents as in farms B and C but also wild boars. In addition, rabbits and bees were reared in the same farm. Furthermore, the quality of the litter could increase the rate of *Campylobacter* in farm A where the litter was a spent litter but moist in contrast to the other farms (66.7%) where litter was a fresh litter but dry. However, some authors reported that spent litter is more bactericidal than fresh litter, but *Campylobacter* might be less able to survive in a dry litter [23]. Moreover, Szalanski *et al*. [24] isolated *Campylobacter* spp. from filth flies present in turkey housing for the 1^st^ time in the USA with a contamination rate of 23.3%. Thus, the existence of house flies in 66.7% of animal production facilities (Farms A and B) may contribute to turkey and human contamination. Nevertheless, the lowest rate of contamination was found in farm B. Despite the presence of significant sources of contamination in this farm; subjects were reared within a plastic greenhouse where only turkeys were raised in contrast to the other farms (A and C) where turkeys were reared after broilers. However, no deduction can be done because no study has reported if the prevalence of *Campylobacter* depends on the type of housing.

In the visited slaughterhouses, thermophilic *Campylobacter* was isolated from all the cecal content samples with a very high prevalence ranging from 80 to 95% (Table-2). However, lower rates were observed in many developed countries like the USA (55%) [9]. According to Jeffrey *et al*. [25], flock contamination by thermophilic *Campylobacter* is most likely reflected by intestinal samples in a slaughterhouse; this suggests that all the farms which provided these positive flocks were highly contaminated by thermophilic *Campylobacter*. Besides, the rearing period that seems decisive for intestinal colonization, carriage of *Campylobacter* spp. could be increased by stressful events involved by transport and non-compliance of both feed withdrawal and rest period that were observed in all the visited slaughterhouses [26].

It seems established that contamination of carcasses during processing occurs directly through the intestinal contents or indirectly through equipment and water [27]. In traditional slaughterhouses and modern slaughterhouse, workers could also be a source of contamination (Table-1). In traditional slaughterhouses, A, B, and C, thermophilic *Campylobacter* was isolated from neck skin samples with an overall prevalence of 37.1%. In these establishments, significant points of cross-contamination during processing represented by scalding, defeathering, and evisceration [28] were absent or done manually. Besides, the absence of scalding step in the visited traditional slaughterhouses (100.0%), defeathering was manual, and carcasses evisceration was performed on a table where the viscera were removed by the workers toward the legs and not toward the head. Therefore, carcasses contamination could not be related directly to the gut content of the same subject, but it could be related to feathers contamination by fecal droppings. As mentioned above, workers can constitute an important source of transmission and dissemination of *Campylobacter* strains in the visited slaughterhouses. However, initial contamination of carcasses or initial source of fecal contamination occurs either directly from gut content or indirectly notably during scalding, defeathering, or evisceration step [28]. By observing the evisceration method in traditional slaughterhouses, we concluded that the first contamination of carcasses was caused by fecal contamination of feathers probably at farms, during transport or at slaughterhouses [29]. Furthermore, to not contaminate themselves first with intestinal contents, workers were very careful at the evisceration step because, unfortunately, they did not wear gloves. All the more so Jacobs-Rietsma has reported that fecal contamination of feathers represents an important source of *Campylobacter* for poultry carcasses [30]. This observation may explain the fact that the prevalence of thermophilic *Campylobacter* was higher in cecal contents than in neck skins (p<0.05).

A high prevalence (96.7%) of thermophilic *Campylobacter* was noticed in modern slaughterhouse D. In this processing plant; neck skin could be contaminated either indirectly or directly. However, various studies have considered that the scalding water represents a significant source of contamination [8,28], but, in our study, where turkey lot was processed just after processing broiler lot, the scalding step may have prevented cross-contamination between flocks. Indeed, scald temperature was at 60°C, and according to Sanchez *et al*. [31], a temperature superior to 53°C does not allow for the survival of *Campylobacter*. Indirect contamination could, however, be related to the slaughtering equipment notably the feather removal machine that may enable recontamination and cross-contamination between carcasses belonging to the same flock [32]. Defeathering operation has been reported as a significant source of cross-contamination because the equipment led to intestinal content expulsion [4]. Furthermore, as reported by Berrang *et al*. [33], except direct contact with the skin, most of *Campylobacter* isolates that are carried into the equipment after defeathering are found on the skin. Direct contamination of neck skins may be related to gut contents which could increase the rate of thermophilic *Campylobacter* in the modern slaughterhouse. According to Franchin *et al*. [32] and Gruntar *et al*. [34], during the evisceration step, the intestinal rupture with extravasations of its content is always possible, and this stage represents the main factor responsible for cross-contamination that can lead to a substantial increase in *Campylobacter* detection during processing.

It is recognized that since the 20^th^ century, the number of *Campylobacter* strains isolated from human samples resistant to E and/or CIP is increasing [35]. Similar rates of antibiotic resistance to CIP (73.7%), E (21.1%) [36], chloramphenicol (0.0%), and GM (0.0%) [37] were reported in USA and Germany. Higher resistance rates than our results were also registered in the USA; 84.2% was recorded to AM, 10.5% to chloramphenicol, and 5.3% to GM [36]. Lower resistance rates than ours were noticed in Germany regarding NA (65.3%), CIP (64.5%), and TE (47.7%) [37]. In Algeria, quinolones, E, TE, and AM are used for therapeutic purposes in poultry flocks. However, the use of chloramphenicol and GM is prohibited [38]. These data may explain the fact that *Campylobacter* strains isolated from slaughterhouses showed only resistance to families of antibiotics that are used in curative treatment. The high rates of resistance and multiresistance, and the frequent resistance patterns that were reported could be related not only to the uncontrolled administration of some antibiotics but also to the extended use (16-20 weeks) of the tested antibiotics in turkey farms. Duration of the breeding period plays a significant role in increasing the number of resistant *Campylobacter* strains to antibiotics [39].

Comparison of antimicrobial resistance rates of thermophilic *Campylobacter* strains isolated from cecal contents and neck skins showed that the difference between these results for each tested antibiotic was not statistically significant (p>0.05). Rates of resistance to NA, E, CIP, TE, and AM for *Campylobacter* strains that were isolated from each slaughterhouse (A, B, C, and D) were significantly different (p<0.05) (Table-4). This deference could be related to several factors such as the region and the uncontrolled administration of antibiotics in flocks. Indeed, a previous study concerning the antimicrobial susceptibility of *Salmonella* isolates in Algeria reported that the use of different antibiotics is widespread and uncontrolled in poultry farms [40]. All the tested strains were resistant to at least one antibiotic (100%), and 96.9% of the isolated strains were multiresistant (resistance to at least two antibiotics). Our results are comparable to those reported by other authors [36,41]. Resistance to CIP and/or E was found in most of the isolates that were multidrug-resistant. As described by D’Lima *et al*. [41], the selection pressure generated by the use of different antibiotics in turkey farms represents the cause of the acquisition of various resistance profiles. Furthermore, except for one drug resistance pattern (AM-CIP), all the antimicrobial resistance patterns that were observed in neck skin isolates belong to drug resistance patterns of cecal content isolates. This observation suggests that all neck skin isolates derived from cecal contents of the same flock and can confirm one more time that there was no cross-contamination between different flocks in the visited slaughterhouses. We suggested this because as we found the same antimicrobial resistance patterns between neck skin and cecal content isolates (more resistance patterns for cecal content than neck skin isolates) (Table-5), it was possible that cecal content isolates of the same flock contaminated neck skins. If the results were different or if we found more resistance patterns for neck skin than cecal content isolates, then this could be related to the presence of other strains from another flock at the time of slaughter. Furthermore, Peyrat *et al*. [42], by observing only resistance rate results, found that resistance rates of *Campylobacter* strain isolated from fecal droppings before slaughter and from neck skins after slaughter were the same. They suggested in their study that the slaughtering process cannot be considered as a source of re-contamination because it did not select strains resistant to antibiotics. Moreover, according to the slaughtering process used in traditional and modern slaughterhouses, contamination of carcasses by other strains of previous flocks with other or new antimicrobial resistance patterns seems impossible.

## Conclusion

Our results revealed that thermophilic *Campylobacter* was isolated with high prevalence in all the sampled turkey flocks where several potential sources of contamination were found. Contamination of turkey carcasses seemed to occur more in a modern slaughterhouse than in traditional slaughterhouse where defeathering and evisceration operations represented significant sources of contamination. Most tested strains exhibited resistance to E and/or CIP. It is alarming because these antibiotics are considered first-choice antibiotics for human campylobacteriosis. As turkey meat production is increasing, human campylobacteriosis can increase too. For this reason, our results suggest that the turkey industry in Algeria could be the cause of a major public health problem through the spread of pathogenic strains of *Campylobacter*, as well as antibiotic resistance. It is more than necessary to prevent *Campylobacter* contamination in Algeria from farm to fork by establishing preventive programs like HACCP along all the poultry production chain. Furthermore, the epidemiological surveillance network of this foodborne pathogen should be established.

## Authors’ Contributions

RB conceived, designed the study and drafted the manuscript under the supervision of TMH. RB and SM designed the experiment protocol under the supervision of MN and TMH. RB collected and analyzed samples. RB and SZ did the statistical analysis. RB and LB revised the manuscript under the supervision of MN and TMH. All authors read and approved the final manuscript.
